# Endoscopic incisional balloon dilation combined with anti‐scarring agents for postoperative esophageal anastomotic strictures

**DOI:** 10.1002/deo2.70062

**Published:** 2025-01-16

**Authors:** Ken Kumagai, Yutaka Takada, Ayaka Sugimoto, Shinjiro Sakagami, Yuri Akioka, Rie Mitani, Akihiro Oshima, Masayuki Kitade, Manami Oshiro, Sonoka Katsuyama, Shogo Ota, Kanna Adachi, Yukari Shimada, Motohito Hayashi, Atsushi Itami, Toshinao Itani

**Affiliations:** ^1^ Department of Gastroenterology Kobe City Nishi‐Kobe Medical Center Hyogo Japan; ^2^ Department of Gastroenterology Medical Research Institute KITANO HOSPITAL, PIIF Tazuke‐Kofukai Osaka Japan; ^3^ Department of Surgery Kobe City Nishi‐Kobe Medical Center Hyogo Japan

**Keywords:** anastomotic stricture, benign stricture, endoscopic balloon dilation, esophageal stricture, triamcinolone acetonide

## Abstract

The management of locally advanced esophageal cancer typically involves esophagectomy; however, postoperative complications, particularly anastomotic stricture, remain prevalent. Anastomotic stricture can severely compromise patients' quality of life by leading to difficulties in food intake. Although endoscopic balloon dilation has become a standard treatment for gastrointestinal strictures, its efficacy is often limited due to the risk of perforation and the potential for recurrent stricture, necessitating multiple interventions. Recent advancements have introduced endoscopic radial incision and cutting methods, which aim to enhance patency by excising scar tissue. We experienced a case resistant to the radial incision and cutting therapy, necessitating further intervention strategies. This report details our experience utilizing a novel technique, endoscopic incisional balloon dilation, which combines endoscopic incisional technique and balloon dilation therapy with anti‐scarring medications, in cases of refractory anastomotic strictures following esophageal cancer resection. We present three challenging cases in which endoscopic incisional balloon dilation yielded significant clinical improvements, alongside supportive literature. Our findings suggest that endoscopic incisional balloon dilation is an effective and safer alternative to conventional methods, capable of addressing complex stricture scenarios while potentially enhancing patient outcomes and quality of life.

## INTRODUCTION

The standard treatment for locally advanced esophageal cancer is esophagectomy. However, a high incidence of postoperative complications, such as anastomotic stricture, has been reported.^1^ Anastomotic stricture can result in impaired food intake, leading to dietary restrictions, nutritional decline, and the potential for food impaction, which increases the risk of aspiration pneumonia and choking. Therefore, it constitutes a significant complication that profoundly affects patients’ quality of life. Surgical revision is often avoided in cases of gastrointestinal stricture, particularly because the underlying cause is frequently related to anastomotic leakage, and the risk of restenosis remains high.^2^ In recent years, endoscopic balloon dilation (EBD) has become a convenient and widely accepted treatment option.^3^ However, EBD carries risks of perforation and a higher likelihood of restenosis, often requiring multiple treatment sessions, especially in cases of long or severely tight strictures.^4^, ^5^

Recently, various endoscopic techniques, such as radial incision and cutting (RIC),^6^ endoscopic radial incision,^7^ and endoscopic incision therapy,^8^ have been introduced for the treatment of esophageal stricture. These techniques are based on a common principle: the use of endoscopic devices to incise and excise fibrotic tissue at the stricture site. Studies have demonstrated that RIC, endoscopic radial incision, and endoscopic incision therapy are all more effective and safer than conventional EBD.^9^, ^10^, ^11^ Among these techniques, RIC stands out for its ability to improve luminal patency by excising scar tissue and has also been utilized in managing strictures following chemoradiation therapy.^12^ However, restenosis frequently occurs even after RIC, and there is a lack of evidence regarding treatment strategies for RIC‐resistant strictures.^13^ Temporary stenting has been proposed as a solution for postoperative strictures, but its high cost, along with risks of dysphagia, ulceration, and spontaneous stent migration, limits its widespread adoption.^14^

This study presents three cases of refractory anastomotic stricture following esophagectomy for esophageal cancer, resistant to conventional EBD and RIC. The strictures were effectively treated with endoscopic incisional balloon dilation (EIBD) and anti‐scarring agents, resulting in favorable outcomes. These findings are discussed in the context of existing literature.

## PROCEDURE

### Radial incision and cutting

**FIGURE 1:**
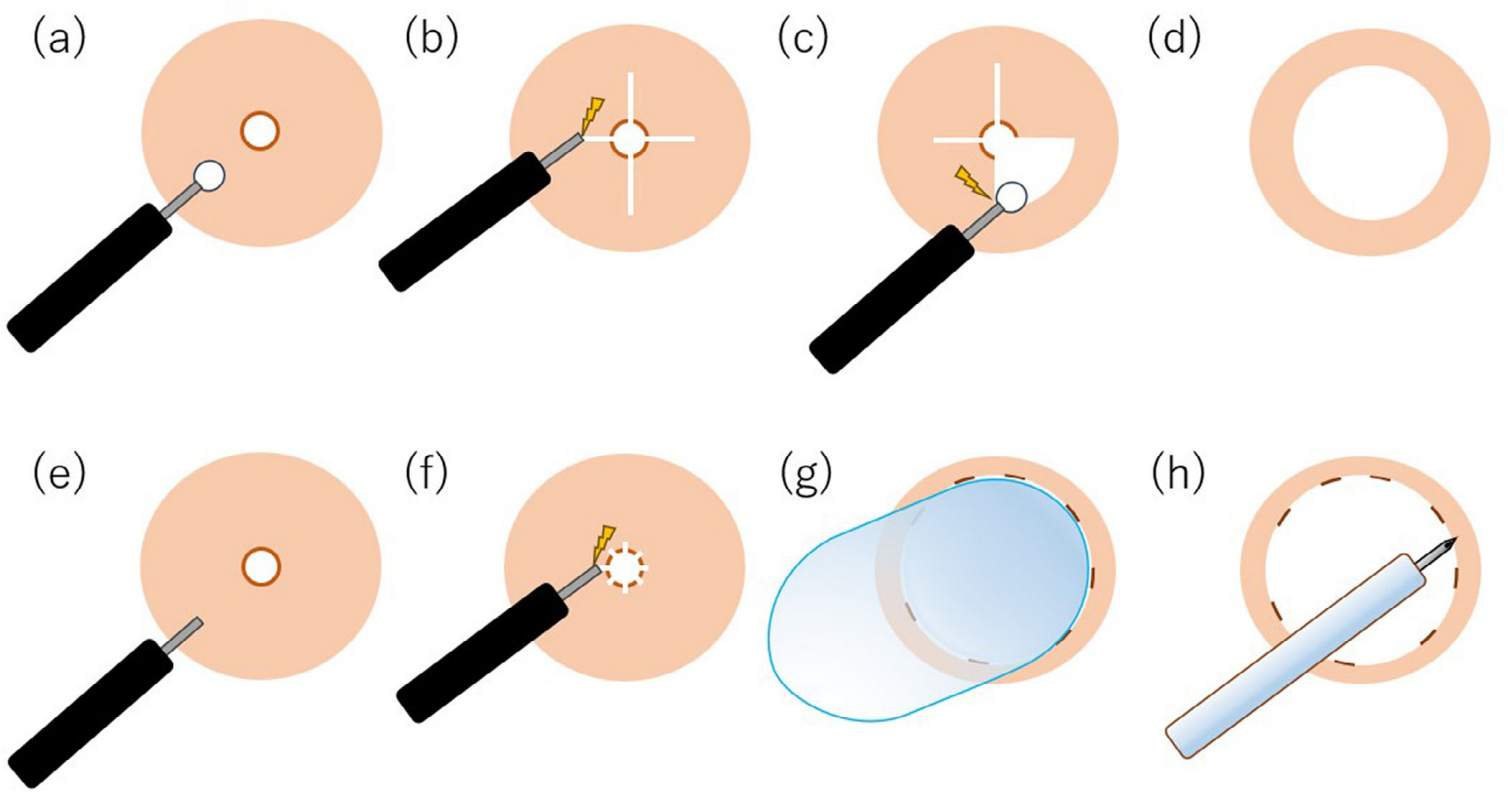
Illustration of radial incision and cutting, along with incisional balloon dilation. (a, b) Deep incisions made using an insulated‐tip knife. (c, d) Excising fibrous tissue. (e, f) Making slight radial incisions using a needle knife. (g) Dilation of stricture using a controlled radial expansion balloon. (h) Circumferential injection of Triamcinolone.

Under conscious sedation, an endoscope was inserted orally, and as previously described,^6^ radial incisions were made at the stricture site using an insulated‐tip (IT) knife (KD‐611L; Olympus Medical Systems). Radial incisions were made at the anastomotic site, taking into account the thickness from both the proximal esophagus and the distal gastric lumen to ensure the incision depth was appropriate and to avoid perforating the gastrointestinal tract (Figure 1a,b). Fibrous tissue was excised using the IT knife, connecting the incision lines (Figure 1c,d). A normal diet was initiated from the evening of the treatment day.

### Endoscopic incisional balloon dilation

Under conscious sedation, an endoscope was inserted orally, and the mucosa at the stricture site was slightly incised radially, with the number of incisions ranging from four to ten, depending on the severity of the stricture, using a sterilized, reusable needle knife (KD‐10Q‐1; Olympus Medical Systems) or a disposable IT knife nano (KD‐612L; Olympus Medical Systems) (Figure 1e,f). When performing incisions with a reusable needle knife, the knife was inserted shallowly below the muscularis mucosa, and cutting was conducted from the outer side toward the inner side of the lumen to prevent deep incisions. In contrast, when using a disposable knife equipped with an insulated tip at its distal end, incisions were performed from the inner side toward the outer side of the lumen. The length of the incision was determined to appropriately match the length of the anastomotic stricture, without excess or deficiency. Subsequently, a controlled radial expansion (CRE) balloon was filled with tap water to dilate the stricture to 18 mm and maintained for 2 min while the mucosal surface was observed through the endoscope (Figure 1g). After deflating the balloon, the passage of the endoscope was confirmed, and triamcinolone (50 mg/5 mL) was injected in 0.5 mL increments around the surrounding mucosa before completing the procedure (Figure 1h). A normal diet was initiated from the evening of the treatment day.

In both procedures, a high‐frequency electrosurgical device was set using VIO3 (ERBE), configured to the Endocut I effect 1.

## RESULTS

The subjects included patients who underwent robotic‐assisted thoracoscopic esophagectomy for esophageal cancer, followed by gastric tube reconstruction and hand‐sewn cervical anastomosis through the retrosternal route. All patients in the cohort experienced postoperative anastomotic leakage, leading to inflammatory anastomotic strictures. While the degree of stricture varied, all cases were resistant to EBD and presented as clinically difficult cases, resulting in an inability to intake food.

### Case 1

**FIGURE 2:**
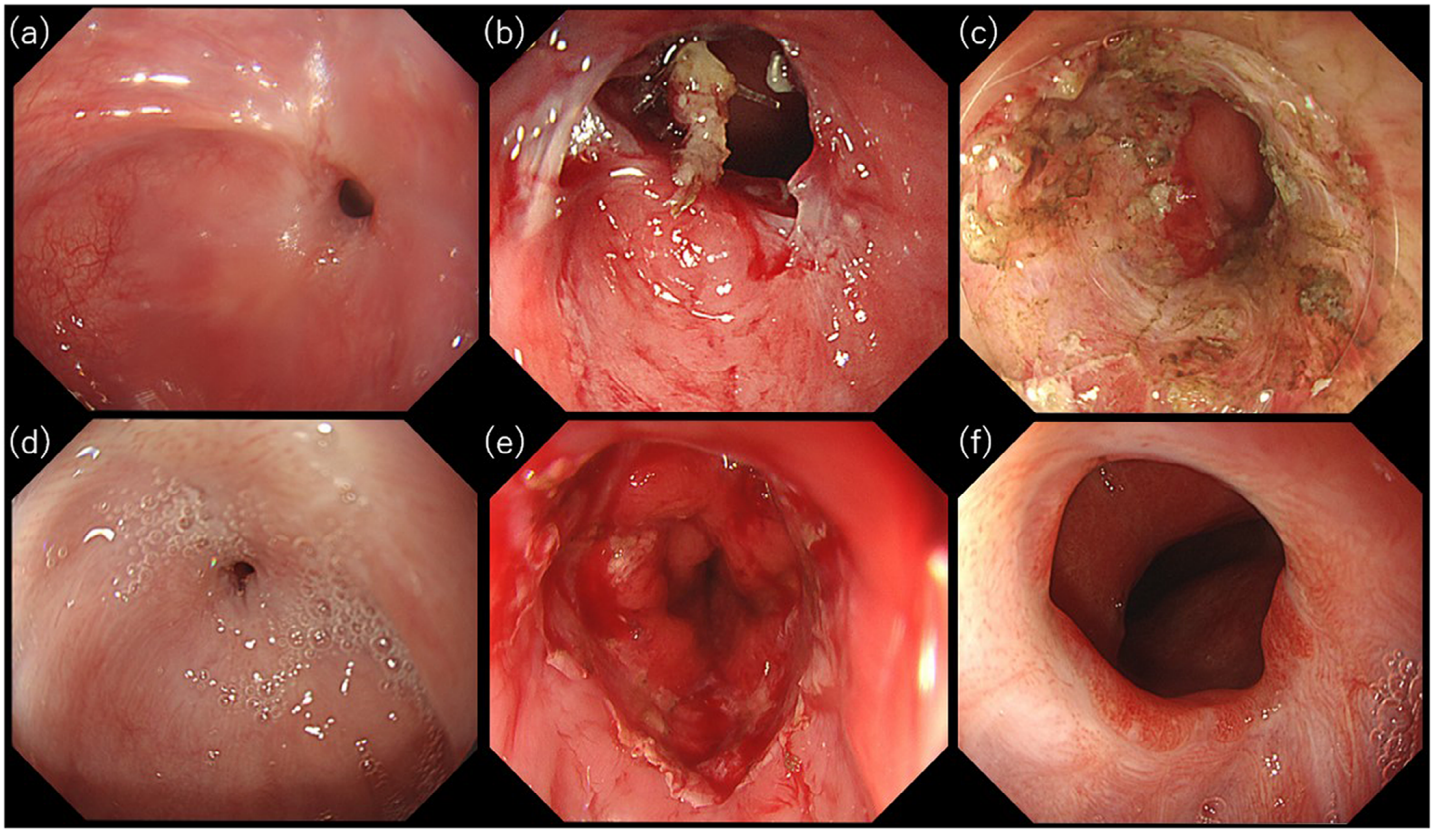
Endoscopic images over time associated with the procedures in Case 1. (a) Pinhole‐line anastomotic stricture. (b) Lacerations following conventional endoscopic balloon dilation. (c) Circumferential ulcer following the first radial incision and cutting procedure. (d) Recurrence of stricture due to granulation tissue formation. (e) Immediately after incisional balloon dilation. (f) Successful relief of the anastomotic stricture after two sessions of incisional balloon dilation.

A 66‐year‐old male presented with dysphagia, and upper gastrointestinal endoscopy revealed pinhole‐like anastomotic stricture (Figure 2a). EBD was initially attempted using a 10 mm CRE balloon; however, the procedure was discontinued due to the laceration of a muscular layer. Subsequently, nine additional EBD sessions were performed, but no improvement was observed. (Figure 2b). Following thorough explanation and consent, RIC was performed as previously described, with the procedure taking 43 min, which included the time from the start of the incision to the successful passage through the stricture (Figure 2c). The patient resumed a normal diet on the night of the procedure, resulting in a dramatic improvement in oral intake. However, 2 weeks later, there was rapid granulation tissue formation leading to restenosis. During the period leading up to the next RIC, EBD was performed to alleviate symptoms. The second RIC was performed 9 weeks after the initial procedure and took 60 min to complete. The patient was prescribed oral prednisolone at a dose of 25 mg daily, and good granulation tissue was observed at the ulcer surface 2 weeks post‐RIC; however, by 3 weeks, restenosis and dysphagia reemerged (Figure 2d). It was concluded that RIC, which creates circumferential ulcers, often results in restenosis during the healing process.

Given the failure of previous treatments to achieve satisfactory results, we proposed creating six shallow radial incisions in the mucosa using an endoscopic knife. This approach was intended to increase the number of weak points and distribute dilation forces more evenly, thereby preventing excessive concentration of pressure. Dilation to 18 mm was then performed using a CRE balloon. As anticipated, no perforations occurred, and the endoscope easily passed through the dilation site (Figure 2e). The patient resumed a normal diet on the same evening and was prescribed oral prednisolone at a dose of 30 mg daily for 1 month. Eight weeks later, although dysphagia recurred, the lumen exhibited significant widening compared to the outcomes of EBD and RIC. Subsequently, an additional EIBD with eight incisions was performed. The patient resumed a normal diet that evening; however, obstructive symptoms reappeared 7 weeks later. A further EIBD using a 20 mm CRE balloon was then performed after six radial incisions were made in advance, followed by the local injection of 5 mg of triamcinolone. The patient resumed a normal diet that evening, and no further recurrence has been observed to date (Figure 2f).

Given the significant therapeutic effects observed with EIBD and the combination of triamcinolone compared to EBD alone or RIC, we proceeded to treat two additional patients with triamcinolone‐combined EIBD, along with tranilast administration, aiming to enhance the effects of triamcinolone through the antifibrotic action of tranilast.

### Case 2

**FIGURE 3:**
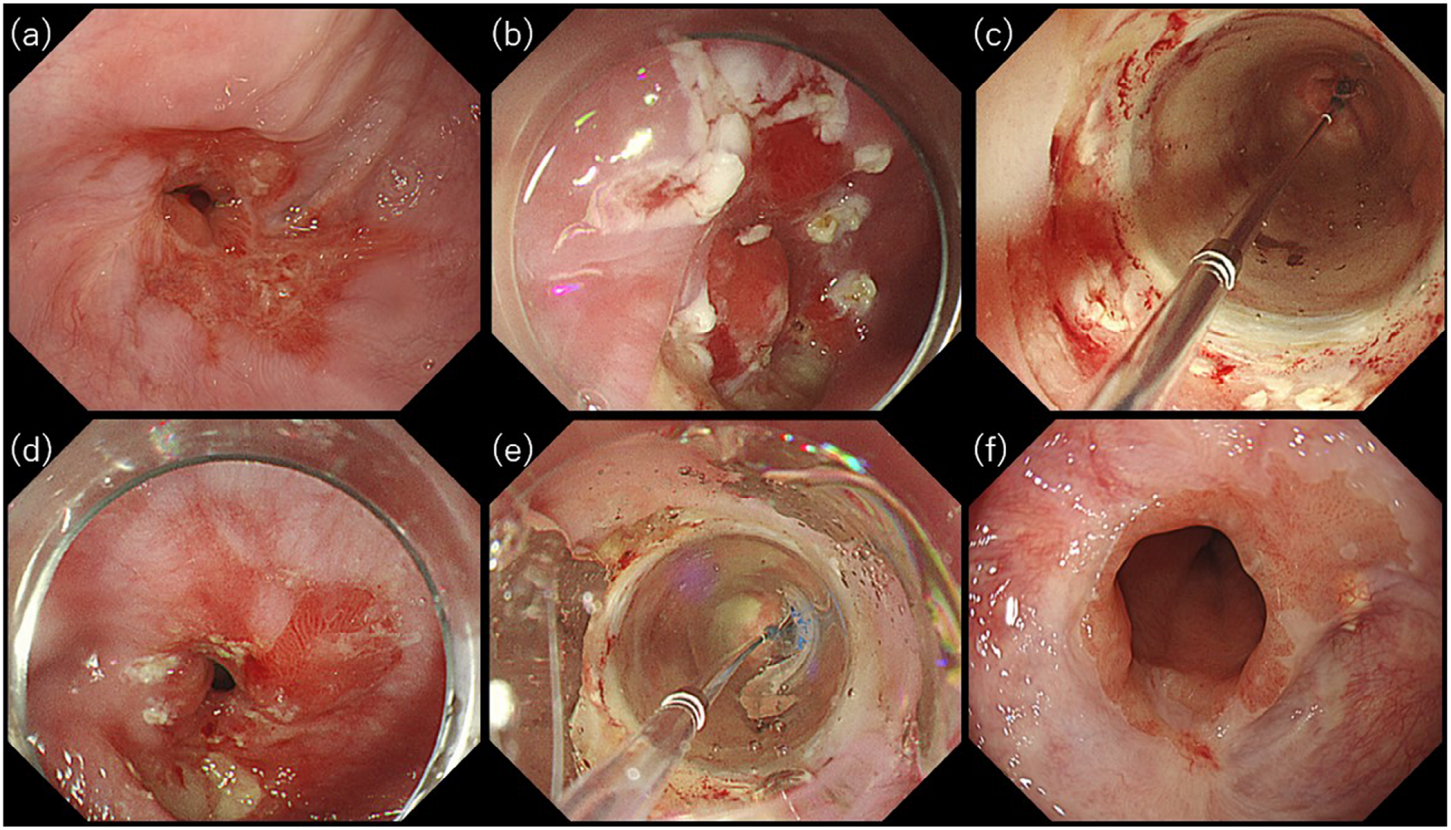
Endoscopic images over time associated with the procedures in Case 2. (a) Anastomotic stricture with severe esophagitis. (b) Slight incisions misplaced circumferentially. (c) Multiple incisions incorrectly placed away from the narrowest part. (d) Slight incisions made circumferentially at the stricture site. (e) Multiple incisions located at the narrowest part. (f) Successful relief of anastomotic stricture after two sessions of incisional balloon dilation.

A 66‐year‐old male presented with dysphagia, and upper gastrointestinal endoscopy revealed anastomotic stricture and reflux esophagitis, attributed to retained food in the proximal lumen (Figure 3a). Three sessions of EBD alone yielded no improvement, leading to the implementation of EIBD with triamcinolone. Initially, due to esophageal deformity and reflux esophagitis, ten incisions were mistakenly placed at a site distinct from the suture line (Figure 3b); balloon dilation to 18 mm and triamcinolone injection was achieved (Figure 3c). However, 12 weeks later, the patient required emergency hospitalization due to poor oral intake, and endoscopy revealed persistent stricture and reflux esophagitis. The endoscope was fitted with a transparent hood to better visualize the anastomotic site, and six radial incisions were made above the anastomotic site before dilation to 18 mm with CRE (Figure 3d,e). The procedure time from the incision to the end of the examination was 10 min. Successful passage of the endoscope was confirmed, and triamcinolone was administered. Subsequently, the patient resumed a normal diet, with excellent improvement observed. A 1‐month supply of tranilast was prescribed for the patient prior to discharge. This patient has since remained symptom‐free for more than 6 months (Figure 3f).

### Case 3

**FIGURE 4:**
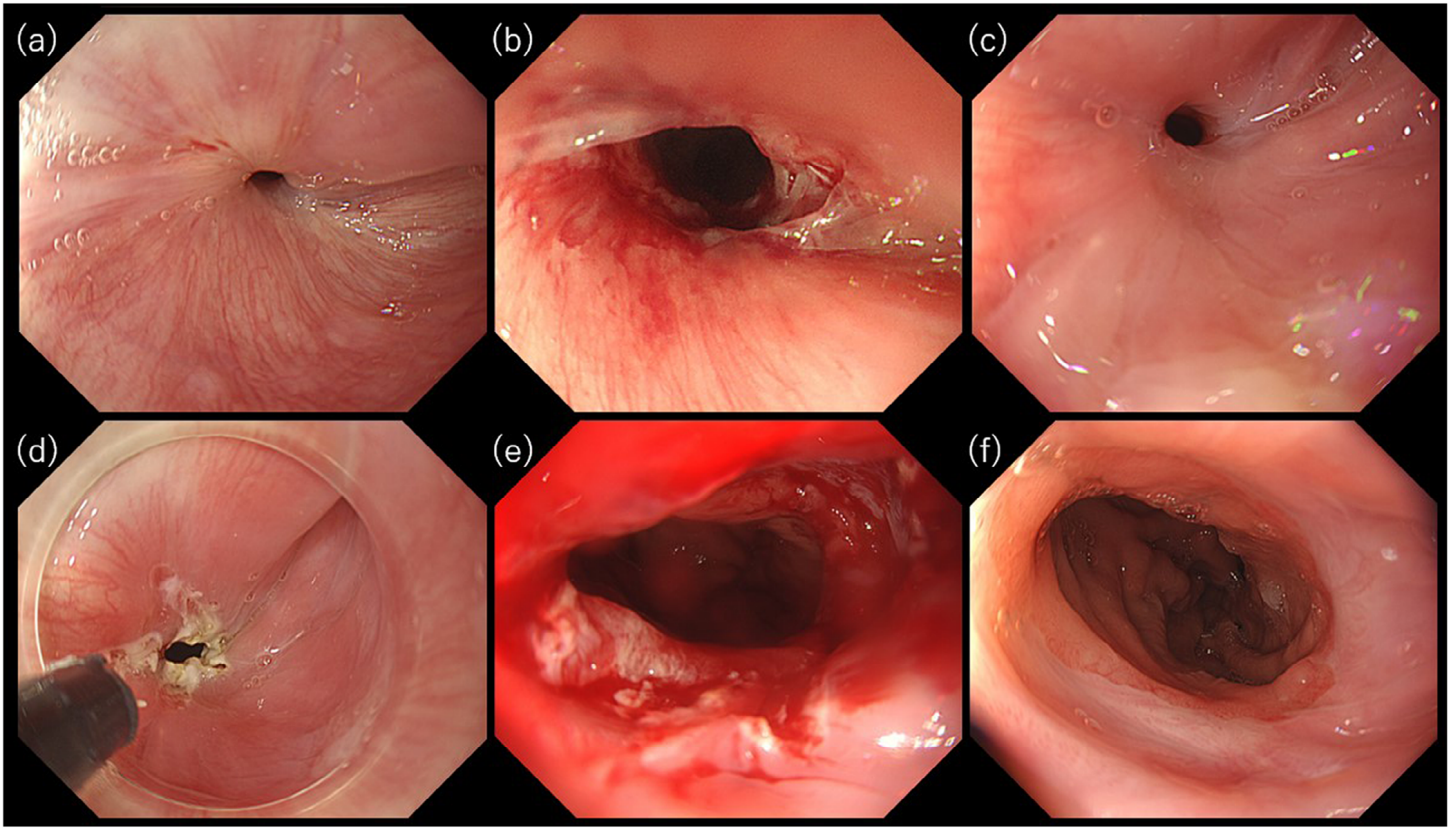
Endoscopic images over time associated with the procedures in Case 3. (a) Pinhole‐like anastomotic stricture. (b) Lacerations following conventional endoscopic balloon dilation. (c) Recurrence of stricture after multiple conventional balloon dilations. (d) Slight incisions made circumferentially at the stricture site. (e) Immediately after incisional balloon dilation. (f) Successful relief of the anastomotic stricture after the first session of incisional balloon dilation.

A 62‐year‐old male presented with dysphagia, which was later confirmed to be caused by anastomotic stricture, with endoscopy revealing a narrow stricture (Figure 4a). Following four EBD sessions with unsatisfactory results (Figure 4b,c), EIBD was performed as previously described (Figure 4d,e). Following four incisions, the anastomotic stricture was dilated to 18 mm, and triamcinolone was injected. The procedure time from incision to the end of the examination, including the time spent observing the gastric tube on the anal side of the anastomotic stricture, was 15 min. The patient resumed a normal diet that evening, and a 1‐month supply of tranilast was prescribed for the patient prior to discharge. A follow‐up endoscopy 1 month later confirmed a significant widening of the anastomosis, with no evidence of stricture (Figure 4f). The patient has subsequently remained asymptomatic for more than 9 months.

## DISCUSSION

**FIGURE 5:**
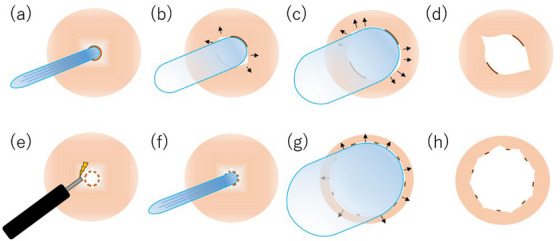
Comparison of ulcer formation and restenosis between conventional and incisional balloon dilation. (a, b) Conventional balloon dilation induces lacerations at fragile sites. (c) Expansion force is concentrated at the laceration points. (d) Deep lacerations disrupt the normal epithelium. (e, f) Incisional balloon dilation creates multiple fragile sites. (g) Expansion force is evenly distributed circumferentially. (h) Normal epithelium is also distributed circumferentially.

We observed the process of restenosis occurring in Case 1 following RIC and concluded that strong granulation tissue formation and contraction were the primary causes of this restenosis. Specifically, we posited that it is crucial to avoid creating a wide ulcer by stripping the mucosa; instead, preserving as much mucosa as possible is essential to prevent re‐contraction. Additionally, retaining the epithelium near the wound is important, as it serves as the source of cells that migrate to the ulcer, while also suppressing the scar contraction process. Furthermore, the uneven hardness of the stenotic area may contribute to inadequate dilation during balloon expansion. When the balloon is inflated beyond a certain pressure, lacerations are initially observed at one or two weak points within the stenosis (Figure 5a,b). These lacerations increase local fragility, causing the expansion force to concentrate on these specific areas, which deepens the lacerations (Figure 5c,d). Consequently, the operator may need to interrupt the procedure without further expansion to prevent perforation of the muscular layer, particularly in cases with a narrow stenotic diameter. This situation often necessitates multiple balloon dilation attempts. By creating several mild lacerations in advance, the inflation pressure of the balloon can be dispersed, allowing for an increase in dilation diameter without leading to perforation (Figure 5e–h). Similar to conventional EBD, EIBD enables real‐time endoscopic visualization of the lumen during dilation, allowing the procedure to be safely terminated before perforation occurs. Given that conventional EBD is routinely performed in an outpatient setting, it is reasonable to consider that EIBD can also be safely conducted in an outpatient setting.

It has been demonstrated that prophylactic administration of triamcinolone through local injection in post‐ESD ulcers in esophageal cancer can suppress collagen formation and reduce scar contraction, thereby preventing stenosis.^15^ The efficacy of triamcinolone has also been shown in the lower gastrointestinal tract.^16^ Moreover, the usefulness of administering the anti‐keloid agent tranilast for preventing stenosis in post‐ESD ulcers has been reported.^17^

By combining these approaches, we successfully addressed refractory esophageal stenosis. In Case 1, we demonstrated that the stenosis, which was difficult to resolve with EBD or RIC, could be effectively treated with EIBD in the same patient. This finding demonstrates that EIBD is highly effective, as it enhances treatment outcomes and reduces the number of required sessions, ultimately lowering overall treatment costs. In Case 2, also involving multiple sessions with the same patient, it was shown that EBD combined with incision at an inappropriate location was ineffective. It is crucial to create multiple incision lines in the stenotic area to effectively distribute the balloon's expansion pressure. This approach not only facilitates the disruption of deep fibrous tissue but also allows the newly formed mucosal defect to be covered by the epithelium before contraction occurs. This process increases the epithelial surface area, which is essential for achieving effective dilation. Additionally, delaying contraction with anti‐scarring medications may further enhance this effect. In Case 3, building on the experiences from the previous two cases, we achieved a significant treatment effect in a single session and demonstrated reproducibility.

The combination of EIBD with triamcinolone and tranilast is thought to enhance efficacy through the following synergistic mechanisms, thereby contributing to the rapid improvement of patient quality of life compared to the incision or EBD alone: it creates multiple mucosal defects to expand the total mucosal surface area without excessively damaging healthy mucosa; it facilitates uniform distribution of balloon pressure, maximizing the diameter of dilation in a single attempt and allowing for further expansion of the mucosal defects; and the combined use of triamcinolone and tranilast inhibits collagen synthesis and fibrotic scar formation,^17^ thereby suppressing scar contraction and preventing luminal re‐narrowing associated with healing. The significant importance of this treatment lies in its potential to reduce the duration of symptoms, such as dysphagia and chest pain, associated with refractory anastomotic strictures of the esophagus, which typically require repeated interventions like EBD and RIC. In the treatment of strictures following subtotal esophagectomy, epithelialization is often completed within 1 month following dilation. In this study, the administration period for tranilast was set at four weeks, and the decision to perform additional EIBD, if necessary, was made 1 month after the initial EIBD based on endoscopic evaluation.

However, there are limitations to this study, including the small number of reports and the lack of subgroup analysis regarding the presence or absence of triamcinolone and tranilast, which prevents us from determining their individual contributions. Furthermore, the optimal number of incision lines to form during incision for maximum efficiency has not been evaluated. In addition, there is no information regarding the efficacy of this treatment compared to EBD and steroid local injection combination therapy, highlighting the need for studies comparing the effectiveness of both approaches. Therefore, further comparative studies regarding the presence of tranilast, the number of incision lines, and the comparison between this treatment and other therapeutic modalities are warranted.

## CONFLICT OF INTEREST STATEMENT

None.

## ETHICS STATEMENT

The research protocol for this study was approved by the Institutional Review Board of Kobe City Nishi‐Kobe Medical Center on October 15.

## PATIENT CONSENT STATEMENT

Informed consent was obtained from all participants prior to their involvement in the study.
